# A Timeline of Clinical Applications of Robots in Cranial Neurosurgery

**DOI:** 10.1002/rcs.70251

**Published:** 2026-11-04

**Authors:** Anjum Aarifa Khanom, Callum Ward, Joseph Davids, Arup Ray, Aprajay Golash, Andrew Alalade

**Affiliations:** ^1^ Aintree University Hospital Liverpool UK; ^2^ School of Medicine University of Lancashire Preston UK; ^3^ Imperial College London London UK; ^4^ Royal Preston Hospital Preston UK

**Keywords:** cranial, innovation, learning curve, neurosurgery, robotics, technology

## Abstract

**Background:**

A robot is a reprogrammable multifunctional manipulator designed to move tools through varied motions. In surgery, robots have been introduced for their tireless operation, precision, and consistency. However, the use of cranial neurosurgical robots remains in its infancy.

**Methods:**

A literature search was conducted in PubMed, Medline and Google Scholar. This search focused on the first clinical applications of robotics in cranial neurosurgery to date. A manual search was also conducted.

**Results:**

Twenty robotic systems were identified. Sixteen were passive devices primarily used for stereotactic neurosurgery. Shared and supervisory‐controlled robots are increasing in prevalence due to technological advances. Eight systems are commercially available, highlighting their success in meeting clinical needs.

**Conclusion:**

It is apparent that the landscape is dominated by stereotactic robots with passive functioning. There is an increasing prevalence of active and shared‐control systems. Advancements in technology, cost and learning curve remain barriers to the scalability of neurosurgical robots.

## 1 Introduction

A robot is a reprogrammable, multifunctional manipulator designed to move material, tools, or specialised devices, through variable programmed motions to perform a variety of tasks, as defined by the Robot Institute of America [1, 2, 3]. Robots have been introduced into industry, and more recently into healthcare and surgery for their indefatiguing nature, accuracy and repeatability [1, 3, 4, 5]. These traits enable their purpose of supplanting and enhancing human effort to the benefit of both patient and surgeon.

The first application of surgical robotics translated experience from the industrial sector into operating rooms. The Programmable Universal Machine for Assembly 200 (PUMA 200) (Advance Research & Robotics, Oxford, CT) was initially an industrial robot [6, 7]. It was modified by Kwoh et al. to define the trajectory of a frame‐based (FB) brain biopsy in 1985, therein crediting the advent of robot‐assisted surgery (RAS) to neurosurgery [6]. The robot demonstrated higher precision accuracy than standard practice at the time but was discontinued due to safety regulations of industrial robots working in proximity to humans [6, 7]. In over 3 decades since then, RAS has had a significant impact on multiple surgical specialties, with the da Vinci Robot (Intuitive Surgical Inc., Sunnyvale, CA) being one of the most popular [8, 9]. This robot has been used to perform over 6 million procedures, with 55,000 units installed in hospitals globally—this tremendous growth can largely be attributed to its utilisation in the urologic, gynaecologic and general surgical specialties [9].

In many ways, neurosurgery is a suitable field for exploring RAS [4, 10]. Complex and delicate neuroanatomy means that there is a necessity to work through narrow corridors to reach critical, deep structures [4]. The subsequent length of surgical cases can leave surgeons prone to undesirable fatigue and tremor—for which robots may prove a useful adjunct. Additionally, the skull presents as a solid density to be fixed as a stationary reference point against the encased soft cerebral structures for registration and planning of procedures and mapping neuroanatomy to a computerised coordinate reference system [4, 10].

These factors both invite, and demand high‐performance robotics. However, robots have yet to be incorporated en masse into cranial neurosurgery, and may highlight how the present supply of surgical robots does not adequately meet the needs of neurosurgical procedures [1].

This paper aims to explore a timeline of surgical robots that have, at a minimum, been used in 1 clinical cranial neurosurgical procedure—regardless of whether they have subsequently been discontinued or marketed commercially. They are ordered and marked by the date of their first reported clinical use within cranial surgery (Table 1). Following this, we aim to assess trends in the purpose of robots that have made it to clinical trials and the reasons for their failures or successes.

**TABLE 1:** Summary of robots that have been used clinically in cranial neurosurgery. For equipment that is commercially available, only the first documented clinical cranial neurosurgical publication is listed. For closed projects, all papers describing its clinical use in cranial surgeries are listed.

Robot (Year of publication) [company]	First published clinical use	Classification		Applied procedures within cranial neurosurgery	Design	Patient cohorts within cranial surgery	Pros	Cons	Current status
PUMA (1985) [advance research & robotics, oxford, CT]	1985	Passive	Supervisory controlled	Stereotaxis Tumour resection	Single arm with 6 DoF; image‐guided trajectory planning; gripping and manipulating retractors and biopsy cannulae	7 patients	Accuracy	Safety	Closed
Minerva (1990) [university of lausanne, lausanne, Switzerland]	1993	Passive	Supervisory controlled	Stereotaxis Needle aspiration	Single arm with 5 DoF; real‐time CT‐guided trajectory planning by operating within the gantry; capable of biopsy, aspiration, drilling, skin incision	10 patients	Reduced operative time; improved workflow	Safety	Closed
MKM (1997) [carl zeiss, oberkochen, Germany]	1997	Passive	Supervisory controlled; tele‐surgical	Stereotaxis Tumour resection AVM resection Microsurgery Transsphenoidal skull‐base	Optical microscope; single arm with 6 DoF robotic manipulator; tool holder enabling stereotactic tool guidance	237 patients	Versatility: Super‐ imposition of 3D anatomical reconstruction onto the microscope optical field minimises skin incision and craniotomy	Bulky; acquisition cost	Closed
Surgiscope (1989) [ISIS robotics, Saint Martin d’Hères, France]	1998	Passive	Supervisory controlled	Stereotaxis Microsurgery	Ceiling mounted single arm with 7 DoF; two primary functions are microscope and biopsy	Indeterminable [commercially available]	Integrable upgrades enable diverse applications	Extends operating time; high purchasing cost and cost of upgrades; lack of mobility	Commercially available
Neuromate (1987) [renishaw, new mills, wotton‐under‐edge gloucestershire, UK]	2002	Passive	Supervisory controlled	Stereotaxis Microsurgery Skull‐base Resection	Single arm with 6 DoF; designed for holding and supporting instrumentation; frameless and frame‐based modes	Indeterminable [commercially available]	Robust; accuracy	Purchasing cost; bulky	Commercially available
NeuRobot (2003)	2003	Passive	Tele‐surgical	Tumour resection Microsurgery	Tele‐surgical manipulation of 3 arms and 1 endoscope through a 10 mm port	2 patients	Dexterity; multiple arms	Bulky; lacks manoeuvrability	Closed
Evolution 1 (2004) [universal robot systems, schwerin, Germany]	2004	Passive	Tele‐surgical	Microsurgery Transsphenoidal skull‐base	Single arm with 6 DoF; tele‐surgical control via joystick; parallel actuator for high payload and accuracy of speed and motion	6 patients	High payload; accurate control of speed and motion through joystick	Bulky; costly; lacks necessary force‐feedback	Closed
CAS‐BH5 (2008)	2008	Active	Tele‐surgical	Microsurgery Tumour resection Haematoma evacuation Stereotaxis	Single arm with 5 DoF; tele‐surgical operation designed for continental distances	10 patients	Versatile	No data on signal latency over long‐distance tele‐surgery	Presumed closed
Pathfinder (2009) [prosurgics ltd., high wycombe, United Kingdom]	2009	Passive	Supervisory controlled	Stereotaxis Transsphenoidal skull‐base Tumour resection	Single arm with 6 DoF; frameless tool positioning using photo‐reflective fiducials	37 patients	Accuracy	Fiducials present as risk for registration error	Closed
Expert (2010)	2010	Passive	Shared control	Microsurgery	Arm rest with 5 DoF; synchronous movement enabled through magnets	13 patients	Reduced tremor; reduced fatiguability	Bulky	Closed
ROSA (2008) [MedTech SAS, montpellier, France]	2011	Passive	Supervisory controlled; shared control	Stereotaxis Stereo‐EEG Epilepsy surgery	Single arm with 6 DoF; haptic feedback capabilities enabling passive supervisory controlled mechanism, or shared‐control use benefitting from tremor filtration	Indeterminable [commercially available]	Accuracy; reduced operation time; improves workflow	Bulky; steep‐learning curve	Commercially available
neuroArm (2002) [IMRIS, winnipeg, Canada]	2013	Active	Tele‐surgical	Microsurgery Stereotaxis	Dual arm with 7 DoF; tele surgical control: MRI‐compatible to work intra‐operatively	35 patients	Dual‐armed; tremor filtration; accuracy	Limited payload; increases operative time	Presumed open
Neuroblate (1999) [monteris medical, winnipeg, Manitoba, Canada]	2013	Passive	Tele‐surgical	Laser thermal coagulation of intracranial tumours	MRI‐compatible robot; tele surgical control of laser with 2 DoF	Indeterminable [commercially available]	Accurate; enables treatment of lesions not amenable to surgery	Purchasing cost; inherent nature of *laser interstitial thermal therapy* requires multiple passes for large tumours, increasing time	Commercially available
iSYS1 (2013) [iSYS medizintechnik, GmbH, kitzbuehel, Austria]	2016	Passive	Supervisory controlled	Stereotaxis	Single arm with 7 DoF; miniature system;	123	Accuracy	Limited series	Open to investigation
Da vinci surgical (2000) [intuitive surgical, sunnyvale, CA]	2013	Active	Tele‐surgical	Skull base tumour resection	3 active instrumentation arms + 1 endoscope holder with 7 DoF; tele‐surgical operation with a variety of compatible instruments for the end effector	6 patients	Versatile; ergonomic master console; tremor filtration; accuracy; multiple arms	Bulky—arms prone to clashing (excluding SP series); purchasing and consumables cost	Open
iArmS (2012) [DENSO, kariya city, aichi prefecture, Japan]	2017	Active	Shared control	Microsurgery Tumour resection	2 arm rests; use force sensors, brakes and encoders to move synchronously	43	Reduced fatiguability and tremor	Not reported	Presumed open
Renaissance/MARS (2011) [mazor robotics ltd., caesarea, Israel]	2019	Passive	Supervisory controlled	Stereotaxis Microsurgery	Single arm with 6 DoF; small hexapod base to affix directly to the skull or mayfield; intra‐operative spatial monitoring using external fiducials	Indeterminable [commercially available]	Small and portable; robust for application	Further data in cranial subjects required	Commercially available
ENDOFIXexo (2015) [AktorMed GmbH, Germany]	2019	Passive	Shared control	Endoscopy	Single arm with 5 DoF; sole function as an endoscope in skull‐base procedures to allow bimanual operation from the surgeon	Indeterminable [commercially available]	Robust	Lacks manoeuvrability; safety concerns	Commercially available
Cirq (2018) [Brainlab, Munich, Germany)	2020	Passive	Supervisory controlled	Stereotaxis	Single arm with 7 DoF; can be affixed to the patient table	Indeterminable [commercially available]	Lightweight; small	Little data in cranial surgery	Commercially available
ExcelsiusGPS cranial solution (2023) [GlobusMedical, Pennsylvania, United States]	2023	Passive	Shared control	Stereotaxis	Single arm with 6 DoF; frame‐based or frameless tool positioning and able to accept a variety of instruments as end‐effector equipped with a variety of guide tubes. CT‐fluoroscopy registration available to reduce radiation exposure.	Indeterminable [commercially available]	Accurate; robust frame‐based approach	Little published data in cranial surgery	Commercially available

Abbreviation: DOF = degrees of freedom.

## 2 Materials and Methods

A literature review was undertaken between January–March 2024 to review studies detailing the use of robotics in neurosurgery. Studies focussing on the clinical application of a robot in cranial neurosurgery were included. Following this, a scoping review of the literature was undertaken to explore those robotic systems further. Surgical robotics used in spinal surgery or systems lacking at least one clinical application of the robot in cranial neurosurgery were excluded. No ethical approval was required for the present study.

To ensure contemporaneity of the review, an updated focused search was performed in January 2026 to identify publications from 2024 to 2025 relating specifically to robotic‐assisted minimally invasive cranial neurosurgery, skull base robotics, orbital robotic applications and novel microsurgical robotic platforms. Particular attention was paid to systematic reviews and first‐in‐human feasibility studies to contextualise recent advances and emerging clinical directions.

## 3 Results

### 3.1 Classification of Robots

Several classifications for surgical robots exist, with the proposition from Nathoo et al. in 2005 being widely used [3, 7]. Three classes were proposed, that is—*supervisory‐controlled, tele‐surgical and shared‐control.*

For *supervisory‐controlled* systems, the surgeon defines a plan pre‐operatively—the robot then only follows this plan during the operation, under the surgeon's supervision [7]. For *tele‐surgical* or *Master‐Slave* robots, the surgeon directly controls the robot through a form of force‐feedback joystick (master) that connects to and controls the robotic tools (slave) at the patient's bedside in real time [7, 10]. *For shared‐control systems*, the surgical instruments are controlled by both the surgeon and the robot who work together synergistically—this is most often employed for steady‐hand manipulation [7, 10].

Additionally, robots are often classified into *passive* and *active* systems. Passive systems are effectively ‘powered‐off’ during the operation—they are positioned using a pre‐operatively defined plan or by the surgeon's physical effort, then locked in place to hold tools statically [3]. Active systems remain dynamic throughout the operation by responding to changes in their environment to varying degrees –effectively remaining ‘powered‐on’ throughout the operation [3].

### 3.2 Timeline of RAS

#### 3.2.1 PUMA 200 (1985)

A passive, supervisory‐controlled system was adapted from industry to aid CT‐guided stereotactic biopsy. The system had a single arm with 6 degrees of freedom (DoF), which positioned a cannula along a pre‐defined trajectory to pass a biopsy needle [6]. It was utilised once more in 1991 to hold a retractor during the resection of 6 paediatric thalamic astrocytomas [11]. It increased accuracy when compared to standard practice at the time [6, 11]. However, due to safety concerns of utility of an industrial robot with surgery, the project was subsequently closed, and the system is no longer used [11].

#### 3.2.2 Minerva (1993)

A passive, supervisory‐controlled system was the first real‐time image‐guided system [12]. By taking successive CT images to position the tool intraoperatively, it aimed to increase accuracy by eliminating error from brain‐shift [13]. The robot arm had 5 DoF, and operated inside the CT gantry [13]. It was built with several compatible tool tips to enable more than stereotaxis—including skin incision and bone drilling—which were trialled in cadavers [13]. The first clinical application was published in 1993, using its aspiration biopsy probe in 2 patients, with a further series of 8 patients in 1995—a decrease in the total procedure time was reported [13, 14]. However, it was discontinued due to its reliance on CT imaging, which exposed the patient to an unnecessary amount of radiation [15]. The system is no longer used.

#### 3.2.3 MKM (1997)

An active, tele‐surgical microscope system incorporating supervisory‐controlled elements—it was built as a frameless stereotactic microscope and later was used for tool navigation [14, 16]. This device comprises of three essential parts: an optical microscope with an autofocus lens, a carrier robotic arm with 6 DoF, and a computer workstation [17, 18]. The product's capabilities were expanded with a tool holder, enabling probe guidance and bone drilling [19]. The microscope could move autonomously to predefined locations, but the tools required manual manoeuvring using a joystick. Frameless registration was enabled using bone or scalp fiducials [10]. The system's first published clinical use was in 1995, on a cohort of 86 patients, to guide a variety of tumour resections [20]. In 1997, a paper published its use in 115 procedures for a variety of indications, and further sequences of 23 stereotactic biopsies and 25 transsphenoidal surgeries were reported by two separate studies, both in 2003 [10, 16, 21]. This project has since been discontinued, with its bulky frame and cost being potential contributing factors [16, 21].

#### 3.2.4 SurgiScope (1998)

A passive, supervisory controlled endoscope system utilised in endoscopic, biopsy or neuro‐navigation procedures [16]. It is a ceiling‐mounted robotic arm with 7 DoF, and can be upgraded by integrating different tools, such as a tool holder for compatible instrumentation [16]. It is often attached to a Leksell model G insertion guide carrier unit (Elekta AB, Stockholm, Sweden), which also acts as a guide for drilling, or catheter passage [22, 23]. It has two primary modes—microscope mode in which it focuses the endoscope's optical axis along a pre‐defined trajectory onto its target and biopsy mode where the probe attachment advances along the pre‐defined trajectory [16]. It remains commercially available [16].

#### 3.2.5 Neuromate (2002)

A passive, supervisory‐controlled system intended for stereotactic and endoscopic procedures [8, 16, 24]. Its main functions are holding, supportive and stabilising instrumentation on pre‐defined axes to improve workflow [8]. It is designed as a single robotic arm which contains 5 joints, enabling 6 DoF. The robot can be used in a FB or frameless manner, where registration is achieved via ultrasound [8, 16]. The former has a reported accuracy of < 1 mm, while the frameless approach increases this to 1.95 ± 0.44 mm [16, 24]. Its commercial availability has afforded this robot a strong literature base, which highlights its robustness and accuracy for its applied procedures [24, 25, 26].

#### 3.2.6 NeuRobot (2003)

An active tele‐surgical system is a three‐armed device (each with 3 DoF) with an endoscope [27]. The end effectors of each arm are micro‐manipulators and at 3 mm in diameter, pass through a single 10 mm port, with an additional 4 mm endoscope [8, 27]. The system has been used clinically in 2 patients—firstly, for the resection of an atypical meningioma in 2003, and then to manipulate micro‐forceps in an ETV in 2012 [27, 28]. No further information has been obtainable since 2012 [8, 28].

#### 3.2.7 Evolution 1 (2004)

A passive, tele‐surgical system designed for endoscopic procedures and tool manipulation [15]. With a hexapod base and parallel actuator, its single robot arm with 6 DoF can assimilate different instruments—including high‐speed drills and endoscopes [8, 16, 29]. The user interface couples a touch screen and joystick to control the tools’ speed and motion [16]. In 2004, the endoscope effector was used in 6 patients for an endoscopic third ventriculostomy (ETV) instead of a handheld endoscope, improving precision targeting and enabling bimanual operation by the surgeon [30]. In the same year, it was used for endoscopic transsphenoidal resections of large invasive pituitary adenomas in 2 patients [31]. Although the surgeries were successful, the reported drawbacks were its lack of sensory feedback and high cost [8, 30, 31, 32]. Furthermore, the parallel actuator made the system bulkier than necessary [16]. The system is no longer open to study and not available for use [8].

#### 3.2.8 CAS‐BH5 (2008)

An active, tele‐surgical system designed for frameless stereotactic cranial procedures over large geographical distances [33]. Like most Master–Slave systems, it is composed of 3 fundamental parts—an operation planning system to plan the procedure, a surgical localization system comprising the robotic arm and tools required for the procedure and a telemanipulation operation system enabling communication between the two [33]. The surgical localization system has a single robotic arm with 5 DoF, with a camera to enable visual registration and navigation, and many compatible surgical instruments [33]. The system had initially been used in 2008 in a series of 10 patients for long‐distance operations over 1300 km using a ‘Digital Data Network’ for pathology such as intracranial malignancies and haematomas [33]. Further clinical cases have not been published since 2008.

#### 3.2.9 Pathfinder (2009)

A passive, supervisory‐controlled system designed for frameless image‐guided tool positioning [34]. The robot has a single arm with 4 joints, resulting in 6 DoF [16, 34, 35]. It accurately locks instrumentation onto a predetermined position that is accepted by the cannula end effector [16, 34]. Frameless registration is enabled by the LED camera on the robotic arm, which registers space by identifying photo‐reflective fiducial markers affixed to the patient's head [34]. The robot achieved accuracy of registration to < 1 mm without a clamp and has been used in a series of 37 patients for a variety of procedures [19, 35]. Concerns were raised with the accuracy of the photo‐reflective fiducials in certain lighting conditions, and the effect of movement on skin fiducials, during phantom studies [36]. This system has since been discontinued due to financial reasons [16].

#### 3.2.10 Expert (2010)

A passive shared‐control system was designed as an arm rest to reduce fatiguability and tremor [37]. This surgical aid was 3‐part—an arm holder, a holder support, and a base [37]. The holder support itself has 5 DoF and lacks electric motors. Instead, the surgeon wears a ferromagnetic wristband under sterile clothing that connects to the electric magnets in the arm holder. 3 magnets are installed within the arm holder, meaning synchronous movement is achieved by magnetic force [37]. The system was used in 13 patients from 2010, and since 2013, is no longer open to study or available for use [37].

#### 3.2.11 ROSA (2011)

A passive supervisory‐controlled system with some shared control manoeuvrability was built for image‐guided stereotactic and non‐stereotactic procedures [16]. The monitor and robotic arm manipulator with 6 DoF are mounted on a mobile base [16]. The surgeon can supervise the robot carrying out pre‐operatively defined tasks, or it can act as a shared‐control system wherein the surgeon can control the instruments but benefit from the increased support. Registration can be carried out with a frame or use fiducial markers for a frameless approach [8]. It can perform tasks such as drilling, use probes, or insert electrodes along pre‐defined axes—with the neurosurgeon introducing these manually through the robot's fixed cannula, or the robot can perform the tasks themselves using its haptic force feedback system [8]. Case series originating in 2011 began being published from 2014, and found the frameless approach to be safe and accurate [38]. The system remains commercially available.

#### 3.2.12 neuroArm (2013)

An active, tele‐surgical system that is MRI‐guided and compatible for a variety of neurosurgical procedures. It is a dual armed system, each arm with 7 DoF, and is controlled at a remote workstation via a joystick. It uses haptic feedback as its force sensor, which enables its high‐level of accuracy [8, 16, 39]. It has been trialled in 35 patients, primarily in tumour resection [39]. The study concedes that surgeon‐only operations enable greater speed and dexterity; however, neuroArm's motion scaling and tremor filtration capabilities combine to enable greater accuracy [16, 39]. However, the cost of materials required to make it MRI‐compatible makes it an expensive system [16]. Clinical cases of this robot have not been published since 2013 [8].

#### 3.2.13 Neuroblate (2013)

A passive, tele‐surgical system designed for laser thermal coagulation of intracranial tumours. Initially AutoLITT in 1999 (Monteris Medical, Winnipeg, Manitoba, Canada) but since rebranded to Neuroblate, the system mounts onto a stereotactic frame that is MRI‐compatible, and the device then deploys a laser which has 2 DoF [40, 41]. The surgeon can remotely modulate the length and degree of rotation of the laser ablation fibre [40, 41]. The system was first used clinically in 2013 in a series of 10 patients with intracranial tumours deemed too high‐risk for open resection [40]. The system remains commercially available [20].

#### 3.2.14 iSYS1 (2016)

A passive, supervisory‐controlled miniature robot designed for frameless stereotactic image‐guided biopsy—its single robot arm has 7 DoF [8, 42]. The arm is mounted on a carbon plate that is placed underneath the patient [42]. The robot aligns its guidance sheath along a pre‐defined axis, and then locks—and then functions as a fixed working channel to enable alignment of tools [8]. Results of clinical cranial work with this robot began being published in 2016 after a series of 16 patients underwent electrode implantation for epilepsy [43].

#### 3.2.15 Da Vinci Surgical (2017)

An active, tele‐surgical system that dominates the surgical robotics designed for abdominal laparoscopy [8, 41, 44]. The ‘Master’ component contains 2 controllers and displays 3D images with magnified views of the surgical field [45]. The ‘slave’ component has 3 active instrumentation arms and a 4th for manoeuvring an endoscope—the arms have 7 DoF and 90 degrees of articulation, mimicking a wrist joint [45]. The most recent series—da Vinci SP—notably allows all instrumentation to pass through a single 25 mm port [46]. When considering the system's application in neurosurgery, a cadaveric study demonstrated that Standard Series' arms clashed when accompanying an endoscope through a keyhole craniotomy, which limited the procedure. In the context of clinical neurosurgical cases, the system has been used for skull‐base surgeries [47]. The first clinical neurosurgical use was in 2017, when a series of 4 successful *trans*‐oral resections of sellar tumours were published [47, 48].

#### 3.2.16 iArmS (2017)

An active, shared‐control system of 2 arm supports, which can be manipulated intra‐operatively as the surgeon rests their arms [49]. The system has 3 modes—[1] Transfer, which supports and follows the surgeon's arms; [2] Arm Holding, which supports the surgeon's arms in a fixed position; [3] Arm Free, which fixes the robot arms in place whilst the surgeon moves separately and independently [49, 50]. All of these movements are enabled through a series of force sensors, brakes and encoders [49, 50]. Designed to reduce fatigue and tremor, the system was used in a series of 43 patients in 2017, primarily for the resection of tumours [49]. The robotic arms achieved the goal of reducing fatigue and tremor.

#### 3.2.17 Renaissance/MARS (2019)

A passive, supervisory‐controlled system designed for image‐guided tool positioning and drilling [41]. It is comprised of the MARS robot and its controller [16]. The robot is small and portable (weighs 250g), equipped with a single arm of 6 DoF, and can be directly mounted onto the patient's skull through its hexapod base, or using a Mayfield clamp [16]. The robot has 4 integrated software programmes to enable its frameless guidance [16]. The use of external fiducials means that it can intra‐operatively monitor its own relative location, reducing the need for intra‐operative imaging [16]. To our knowledge, the first published clinical series of this robot being used for cranial applications was published in 2019 despite its CE mark for cranial procedures in 2011—the system remains commercially available [41, 51].

#### 3.2.18 ENDOFIXexo (2019)

A passive, shared‐control endoscope holding system, with a 3‐segment single arm consisting of electromagnetic locks that enable 5 DoF [46, 52]. Previously branded as SOLOASSIST, it is designed specifically to hold an endoscope that enables surgeons to work bimanually and reduces tremor from an assistant manually holding an endoscope throughout the length of an operation [53, 54]. The endoscope must be moved manually using the joystick and does not have its own navigation system. Concerns were raised regarding safety when moving the system intra‐operatively [46]. During preclinical cadaveric testing, it demonstrated its ability to reach all anatomical sites from an endonasal approach including the frontal sinuses, and was clinically trialled in 2019 in a series of 18 skull base procedures with success [46, 54]. The system remains commercially available.

#### 3.2.19 Cirq (2020)

A passive, supervisory‐controlled system designed for frameless stereotactic biopsy. The system is a singular robotic arm with 4 joints enabling 7 DoF [53]. The base has an integrated computer system, and attaches easily to the side of operating tables, meaning that it virtually lacks a footprint within a theatre room [53, 55]. The ability to attach a variety of tools to the end effector means it can also be used in cranial biopsy and to position instruments statically [55]. Registration is achieved using either skin or bone‐screwed fiducials [55]. The first published clinical example of the robot being used in cranial procedures was published in 2020, in a series of 14 paediatric cases for brain tumour biopsy [55]. The system remains commercially available.

#### 3.2.20 ExcelsiusGPS Cranial Solution (2023)

A passive, supervisory‐controlled system modified for FB or frameless cranial stereotactic procedures. Featuring a single robotic arm with 6 DoF, FB navigation is achieved by using the additional cranial specific Patient Stabilisation Stand, which is fixed using Mayfield clamps or similar [18]. Despite being commercially available for 2 years, the only published paper exploring its cranial applications clinically was published in 2023, in which 5 patients underwent FB stereoelectroencephalography (SEEG) [18]. The robot was compared with others used for adult SEEG, to which the authors concluded there were no significant differences in mean operative time, electrode placement accuracy, or fiducial registration error [18]. The system remains commercially available.

#### 3.2.21 Recent Advances 2024–2025

Since completion of the primary literature search, several notable publications have further advanced the field of cranial robotic neurosurgery. A 2025 systematic review focussing on robotic‐assisted minimally invasive cranial neurosurgery highlights expansion into skull base, intraventricular and keyhole approaches, while emphasising that high‐level comparative outcome data remain limited [56, 57]. Recent feasibility studies have demonstrated robotic posterior fossa skull base surgery and robotic‐assisted orbital tumour resections, illustrating the growing technical reach of robotic platforms in anatomically constrained regions. Collectively, these studies suggest a transition from purely stereotactic assistance towards microsurgical augmentation; however, most systems remain adaptations of platforms originally designed for other surgical specialties rather than purpose‐built cranial microsurgical robots [57].

#### 3.2.22 Medtronic Stealth Autoguide

A passive, supervisory‐controlled robotic guidance arm integrated within a neuronavigation platform was designed primarily for cranial biopsies and electrode placement. The system functions as a rigid guidance tool that locks onto pre‐planned trajectories following image registration. Its compact bedside‐mounted configuration minimises theatre footprint and integrates seamlessly into existing stereotactic workflows. Clinical reports demonstrate high targeting accuracy comparable to other stereotactic robotic arms, supporting its adoption in biopsy and functional procedures [17].

#### 3.2.23 Symani Surgical System

An active, tele‐manipulated microsurgical robotic platform designed for supermicrosurgery. The system features motion scaling and tremor filtration, with articulated wristed micro‐instruments capable of performing sub‐millimetre anastomoses. Recent reports describe its application in cranial vascular procedures, including STA–MCA bypass and robotic‐assisted scalp reconstruction. Although its enhanced degrees of freedom exceed those of the human wrist at the microscopic scale, ergonomic integration into deep cranial corridors remains a limitation [58].

### 3.3 Comparative Analysis by Neurosurgical Subspeciality

Robotic systems may be more meaningfully classified according to the procedural domain rather than traditional supervisory/tele‐surgical/shared‐control categories. Stereotactic and functional procedures benefit most from trajectory‐based robotic arms, where submillimetric accuracy and workflow standardisation are paramount. Skull base and endoscopic procedures require enhanced articulation within confined anatomical corridors, and current systems face constraints related to arm collision and restricted working angles. Microsurgical and cerebrovascular applications aim to augment fine motor precision through tremor filtration and motion scaling; however, validated superiority in clinical outcomes remains limited. This highlights that robotic success is strongest in trajectory‐based interventions, while fully tele‐manipulated cranial microsurgery remains developmental [59].

### 3.4 Design Constraints for a Dedicated Cranial Microsurgical Robot

No current platform fully satisfies the combined requirements of cranial microsurgery: microscopic precision, multi‐degree articulation within narrow corridors, minimal theatre footprint, ergonomic integration, and imaging compatibility. The human wrist provides approximately six functional degrees of freedom within a compact anatomical structure; robotic systems must replicate or exceed this articulation without increasing bulk. MRI compatibility restricts material selection and increases cost, while miniaturisation of actuators with adequate force fidelity remains an engineering challenge. Furthermore, current haptic technologies do not yet replicate the fine force discrimination required for vascular dissection. These constraints explain why stereotactic robotics has matured more rapidly than tele‐manipulated cranial microsurgery [60].

## 4 Discussion

This review has detailed 20 robots which have been used clinically in cranial neurosurgical procedures since 1985 (Figure 1). Twelve out of 20 systems were introduced as supervisory‐controlled systems, demonstrating that the bulk of RAS within neurosurgery is advanced by stereotactic applications. However, time has certainly demonstrated that tele‐surgical, shared control and active systems are becoming more prevalent as technology develops. Automation of simple tasks, increasing accuracy, reducing tremor, fatigue and operation time remain key objectives.

**FIGURE 1:**
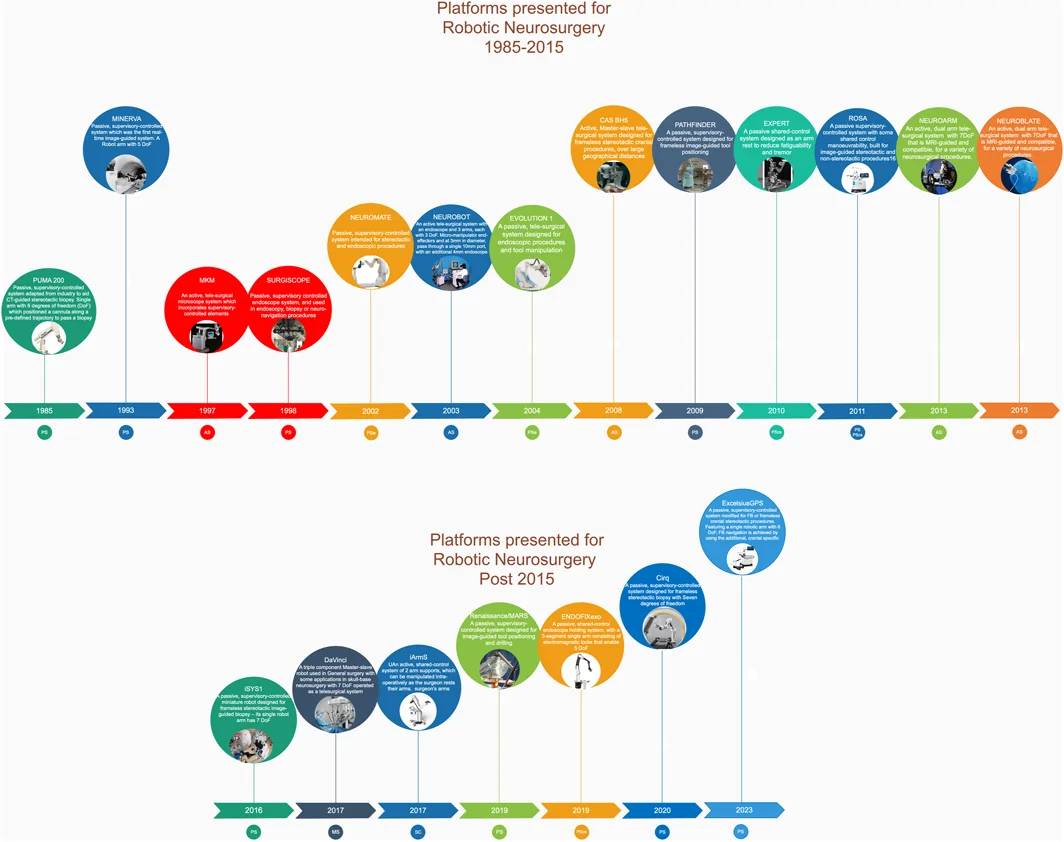
Platforms presented for robotic cranial neurosurgery (1985–2023).

Eight of the presented systems (SurgiScope, Neuromate, ROSA, Neuroblate, Renaissance, ENDOFIXexo, Cirq, ExcelsiusGPS) are commercially available with clinical applications in cranial neurosurgery. As less than half of the presented models have achieved commercialisation, it is important to consider factors which enable versus impede this. Key stakeholders in the conversation on developing technologies remain capital investment firms. One lens applied to forecast the maturity, adoption and social application of technologies is the Gartner Hype Cycle [21]. The theory states 5 stages of development are required over time for a technological innovation to reach mainstream visibility and acceptance.

In the context of surgical robotics, the first stage innovation trigger is, in part, owed to neurosurgery, with utilisation—and failure—of the PUMA 2000 [21]. Following this initial success story in 1985, the majority (6 out of 8) successful commercially available robots presented in this study became successful after the 2010s—demonstrating both the second stage peak of inflated expectations and the third stage trough of disillusionment [21]. With the application and discontinuation of the PUMA 2000, a surge of interest and investment resulted in many early years’ robots that did not meet market expectations, and these failures tempered expectations on robotic applications unique to cranial neurosurgery. In particular, complex neuroanatomy, deep anatomical structures requiring precision with narrow corridors, and limited visibility. These failures resulted in the fourth stage slope of enlightment for stakeholders who are willing to climb the learning curve and meet the nuanced demands of cranial neurosurgery [21]. As both technology innovation companies and surgeons collaborate on an ideal, but realistic product, iterations of high‐performance robots are achieving success more often than failure. This is demonstrated by all 4 of the most recent robots presented in this study successively reaching commercialisation—a fifth stage plateau of productivity [21].

The inference is that the world of surgical robotics within cranial neurosurgery is not only firmly established but also expanding. Widestream acceptance of the presented robots will only result in further refinement—refinement of both individual products, but also of surgical robotic integration as a whole. The discussion, remains on what is required for safe, and superior integration of these robots into practice, when compared to human‐only surgery.

Therefore, an increasingly prevalent topic of literature is deciding on internationally accepted procedural standards for surgical robotics—particularly, on how to measure surgical outcomes, operating room integration, training and learning curves. Indeed, the International Electrotechnical Commission (IEC) and International Organisation for Standardisation (ISO) have developed standards for the basic safe integration of robotics across surgical specialties. Additionally, robots are both tested and improved by regulatory bodies such as the Federal Drug Agency (FDA) and the Medicines and Healthcare products Regulatory Agency (MHRA). However, safe integration is not equivalent to superior application. Despite commercial availability, the literature base for these 8 systems in the context of cranial neurosurgical procedures remains difficult to build, One reason for this is that human and robot decision making—and therefore, outcomes—are not analogous. Human surgeons operate by qualitative decision making, such as using their cognitive judgement and subjective experience. In contrast, robots function via systems and quantitative measures. One potential avenue to overcome this difference in outcome measures is the use of machine learning and artificial intelligence in which robots with pre‐programmed algorithms ‘learn’ and individual surgeon's techniques and translate this into ‘decision‐making’ real‐time—in this scenario, paired outcome data between robots and the surgeon may be extracted. However, there is little to no proof that AI can yet quantify a human's cognitive decision making, or identify the critical task of RAS operations across any surgical specialty—further research into this is required [61]. Secondly, even if the operating mechanism were analogous, this would require widespread availability of robots to not only integrate into operating theatres but also to learn on, and subsequently teach. The financial, administrative and procedural implications of this are manifold and remain unique to countries, institutions and even individual surgeons without international standards.

These discussions highlight that commercialisation of a robotic model is not, in isolation, a testament to reaching the plateau of productivity. Outcomes still require validation. The pharmaceutical industry exemplifies a rigid validation process of early drug discovery, pre‐clinical trials and clinical trials prior to regulatory approval. Although equivalents for surgical robotics development have been proposed, they remain far from international practice [62].

In addition to economical and procedural considerations for robots that have achieved commercialisation, it is important to revisit those that did not make it to the market. Products that fail in the third stage trough of disillusionment of the Hyper Cycle teach us about the remaining development limitations and difficulties in reaching consistent productivity.

### 4.1 Development Limitations

#### 4.1.1 Advancements in Technology

Neurosurgical operations demand uniquely high‐performance robotics. Tele‐surgical systems at a minimum require an endoscope with high‐quality visual feedback, in addition to multiple manipulators with highly sensitive force‐feedback capabilities—all tunnelled through a narrow corridor. This is not yet available commercially. Presently, the technology required to simulate a convincing environment using haptic feedback on the microscopic level required for neurosurgery does not exist; however, it is an important area of research [3]. There is the supra‐added difficulty of quantification of adequate haptic feedback in clinical trials, when this will vary greatly depending on individual surgeon's preference and experience.

#### 4.1.2 Cost

It is estimated that $10 million of venture capital is required for the production of a functional prototype [8]. The development of a robot to meet neurosurgical operation's needs, gain pre‐market approvals and patents, and provide evidence of superseding current practice in clinical trials inflates figures and timescales in an unpredictable and exponential manner. The end‐product is often costly in terms of purchase(s), operational and consumable costs. This affects the scalability of all surgical robots and was the limiting factor for many systems presented in this review [63]. A more practical approach will be to focus on the production of models which present as cost‐effective solutions—for example, with simple disassembly and maintenance or low‐cost consumables [3, 8, 63].

### 4.2 Integration Difficulties

#### 4.2.1 Robot Size

Theatre footprint can hinder optimal workflow and potentially, safety, when considering mass. ‘Bulk’ is a disadvantage of many proposed models—the Evolution 1 was an example wherein the actuator increased bulk to deliver a high payload capacity and accuracy unnecessarily. The implications of accommodating such devices must be carefully considered, with one of the biggest barriers being the size of the operating rooms. Miniaturised devices, or those that clamp to bedsides and stands, incorporate more seamlessly.

#### 4.2.2 Learning Curve and Training

Robotic systems must supercede and/or enhance the surgeon's effort. If the system lacks an intuitive interface and requires increased effort to operate the system, the robot fails to fulfil its fundamental purpose. Notably, systems such as ROSA have become successful despite a steep learning curve, demonstrating that a willingness to learn and train robotics is necessary for the expansion of RAS [8].

The evolution of robotic systems in cranial neurosurgery reflects a broader technological pattern: robotic maturation occurs most rapidly when procedural geometry is linear, repeatable, and image‐defined. In contrast, procedures requiring dynamic tissue interaction, continuous bimanual dexterity, and tactile discrimination have progressed more slowly. This distinction provides a unifying explanation for the dominance of stereotactic systems and the relative immaturity of tele‐manipulated cranial microsurgery.

Importantly, the absence of a dedicated cranial microsurgical robot should not be interpreted as technological failure, but rather as evidence of uniquely demanding biomechanical constraints. The cranial cavity presents a rigid, non‐compliant, millimetric workspace where instrument collision, visual obstruction, and surgeon ergonomics intersect. Any viable robotic solution must therefore integrate compact actuation, expanded degrees of freedom, real‐time collision modelling, and refined haptic interfaces within a footprint smaller than the human hand.

From a translational perspective, this reveals a critical inflection point for the field. Current systems successfully augment precision in trajectory‐dependent tasks, yet the next paradigm shift will require re‐engineering of scale, materials, and control architectures specifically for intracranial manipulation. The future of cranial robotics will likely depend on micro‐actuated wrists, soft robotics integration, advanced force‐sensing technologies, and surgeon–engineer co‐development models that prioritise operative workflow compatibility.

Furthermore, economic and workflow considerations must be acknowledged. Adoption of robotic platforms is influenced not only by technical capability but also by theatre integration time, capital expenditure, learning curves, and demonstrable outcome benefit. Without clear superiority in safety, efficiency, or long‐term patient outcomes, widespread diffusion of complex microsurgical robotics will remain limited.

Taken together, the present analysis suggests that cranial robotics is entering a consolidation phase. Stereotactic systems have reached relative technological maturity, whereas corridor‐based and microsurgical platforms remain in developmental stages. Bridging this divide will require innovations that transcend incremental navigation improvements and instead address the fundamental mechanical embodiment challenges unique to cranial surgery.

## 5 Conclusions

The incorporation of RAS within clinical neurosurgery remains in its infancy. Whilst weight is still applied to stereotactic assistants, the landscape of robots is evolving, with a notable gap for a tele‐surgical system suitable for neurosurgery. Our timeline demonstrates that there are persistent efforts to incorporate RAS for the benefit of the neurosurgeon and patient. Although robotic assistance in cranial neurosurgery has expanded considerably, its application remains predominantly confined to stereotactic domains, with true microsurgical integration still in developmental stages.

## Author Contributions

**Anjum Aarifa Khanom:** investigation, writing – original draft, writing – review and editing. **Callum Ward:** manuscript (re‐drafting). **Joseph Davids:** visualisation, writing – review and editing. **Arup Ray:** supervision. **Aprajay Golash:** supervision. **Andrew Alalade:** conceptualisation, writing – review and editing, supervision.

## Funding

The authors have nothing to report.

## Ethics Statement

The authors have nothing to report.

## Consent

The authors have nothing to report.

## Conflicts of Interest

We declare that none of the authors have any active consultancies, shareholder positions, or financial interests in any of the companies affiliated with the robotic systems discussed in this article.

## Permission to Reproduce Material From Other Sources

No materials were reproduced from other articles for this study.

## Data Availability

Data sharing not applicable to this article as no datasets were generated or analysed during the current study.
